# Energy Balance and Dietary Intake in Young Rugby Players during a Pre-Season Micro-Cycle: A Cluster Analysis

**DOI:** 10.3390/nu16172863

**Published:** 2024-08-27

**Authors:** Maher Souabni, Giovanna C Del Sordo, Freddy Maso, Paul Peyrel, Clément Maviel, Fabrice Vercruyssen, Pascale Duché, Oussama Saidi

**Affiliations:** 1 Laboratory Youth-Physical Activity and Sports-Health (J-AP2S), Toulon University, F-83041 Toulon, France; maher.souabni.phd@gmail.com (M.S.); clement.maviel@gmail.com (C.M.); fabrice.vercruyssen@univ-tln.fr (F.V.); pascale.duche@univ-tln.fr (P.D.); 2 Interdisciplinary Laboratory in Neurosciences, Physiology and Psychology: Physical Activity, Health and Learning (LINP2), UFR STAPS, Paris Nanterre University, F-92000 Nanterre, France; 3 Psychology Department, New Mexico State University, 1780 E University Blvd., Las Cruces, NM 88003, USA; delsordo@nmsu.edu; 4 Rugby Training Center of the Sportive Association Montferrandaise, F-63100 Clermont-Ferrand, France; fmaso@asm-omnisports.com; 5 Department of Kinesiology, Laval University, Quebec, QC G1V 0A6, Canada; paul.peyrel.1@ulaval.ca; 6 Quebec Heart and Lung Institute, Laval University, Quebec, QC G1V 0A6, Canada

**Keywords:** sport nutrition, dietary intake, energy balance, body composition, adolescents, rugby

## Abstract

Rugby players must develop excellent levels of conditioning during adolescence. However, this pivotal period of life is also characterized by a surge in biological growth, which further increases the energy and nutritional requirements of this population. This study examined within-individual differences in energy intake (EI) and energy balance (EB) of 46 young rugby players during a pre-season micro-cycle. Two clusters were identified with significantly different characteristics and EB states, suggesting that young rugby players adjust their EI to match their body composition goals. The first cluster is characterized by players with a low body fat% (12.87 ± 2.53). They had a positive EB (330 ± 517 kcal), suggesting a goal of increasing muscle mass. Conversely, the second cluster is characterized by a higher body fat% (23.1 ± 1.6, *p* < 0.005) and reported a negative, lower EB (−683 ± 425 kcal, *p* < 0.005), suggesting a goal focused on reducing fat mass. Although our study provides more optimistic results than previous ones regarding the high risk of inadequate EI in young rugby players, we emphasize the importance of rigorous nutritional support, especially for players aiming to lose weight, to avoid severe caloric restriction, as well as the downstream effects of such practices on their nutritional status, given the higher risk of macro- (e.g., CHO < 6 g/kg/d) and micronutrient (e.g., iron < 11 mg/d, calcium < 1300 mg/d, vitamin D < 5 mg/d) deficiencies.

## 1. Introduction

Rugby is a team-based invasion sport that combines intermittent bouts of both low and high intensity exercise and involves various physical tasks that require significant strength, endurance, and agility, including activities such as running, tackling and lifting opponents [1]. In order to progress to the senior professional level in this demanding sport, young rugby players must develop excellent levels of fitness and conditioning during adolescence [2]. However, this pivotal period of life is also characterized by a surge in biological growth and maturation that further increases the energy requirements of this population. Therefore, young male rugby players need an outstanding energy intake, along with appropriate macro- and micronutrients intakes, to meet the demands of their sport training and to optimize the physiological changes occurring during adolescence.

Limited research focused on energy intake and energy balance in young rugby players especially during the pre-season period [3,4,5,6,7]. Some of these studies are confounded by the use of traditional dietary assessment tools (i.e., the Australian Eating Survey [3], a four-day food diary [4] and a food frequency questionnaire [8]). However, self-reported dietary assessment methods (i) typically report substantial errors of both validity and reliability [9], and (ii) have not been robustly validated for use within athletic populations, particularly younger ones [10]. Therefore, it is crucial to evaluate the energy intake (EI) using more accurate dietary assessment tools. Moreover, to the best of our knowledge, only Costello et al. (2018) reported the energy balance (EB) in young rugby players during the pre-season period [5]. Despite their high energy intake (almost 4000 kcal·day^−1^), the players reported an energy consumption that was lower than their expenditure, leading to an average negative energy balance (−389 kcal·day^−1^). Accordingly, the authors emphasize the need to be particularly vigilant about young rugby players EI, especially during periods of intensive training. However, while this study offers robust assessments of both EI and energy expenditure, it is important to note the limited number of participants (*n* = 6) and the large within-individual differences found in the EB [5].

In fact, there are two broad playing positions in rugby, namely forward players (FW), including props, 2nd row players, and loose forwards, an adjustable such as hookers, scrum-halves, and stand-offs, and backs (BW) comprising wingers, centers, and fullbacks. It has been reported that these positions demand different physical conditionings and nutritional needs [1,11]. In this context, a recent cohort of 291 players, of which 130 (69 FW and 61 backs) had played international-level rugby and the remaining (94 FW and 67 backs) at elite club level, had their anthropometric and performance data analyzed over a 20-season (from the 1999–2000 season to the 2018–2019 season) period in the UK [11]. Results showed a highly significant variation by position in every anthropometric and performance measure, with FWs being heavier than BWs, and BWs faster than FWs. Importantly, this variation by position has been noticed since adolescence in junior rugby players [12]. Accordingly, most of the studies evaluating the nutritional status of professional rugby players focused on differences in dietary intakes according to playing position. Although playing position demands may differ between FWs and BWs resulting in different energetic and nutritional requirements, we draw attention to the fact that athletes’ nutritional strategies may also depend on weight and body composition goals for each player [13]. This may be even more evident in developing rugby players during the pre-season, when physiological and body composition adaptation is targeted [14]. However, no studies on the potential association of EB with the playing position nor body composition were reported in this population. Considering that optimizing EB and dietary intakes may be important to health and performance in young athletes, it would be relevant to identify the EB within-individual differences and nutritional strategies in different players subgroups. Additionally, it is crucial to note that international guidelines have suggested thresholds for macro- and micronutrients, below which there are potential health risks for individuals [15,16]. Therefore, given that athletes may have different individual nutritional strategies yet display collective trends, we performed a cluster analysis in this study in an attempt to examine energy balance and dietary intakes according to playing position, weight, and body composition. Our primary objective was to assess the EB and dietary intake of young rugby players during a pre-season training week. Secondary objectives included the assessment of macro- and micronutrient intakes. We hypothesized that athletes will not meet international recommendations and will adopt different nutritional strategies according to their body composition. 

## 2. Materials and Methods

### 2.1. Participants

Forty-six male adolescent rugby players engaged in the under-18 national categories (late- or post-pubertal: Tanner stages 4 or 5) participated in this study. They were in good health and were not part of a dietary restriction program. The athletes did not consume tobacco, cannabis, or alcohol. The study protocol was conducted at the rugby training center of the Sportive Association Montferrandaise, Clermont Ferrand, France. Participation in the study was voluntary; parents and young rugby players were informed of the purpose of the study and could withdraw at any time. Information and consent forms were distributed to parents and young rugby players before the study began. This research was conducted in compliance with the Declaration of Helsinki’s ethical standards and received prior approval from the relevant Institutional Ethics Review Board (IRB-00012476-2021190394).

### 2.2. Procedure

At baseline, participants were assessed for anthropometric and body composition in the morning, in a fasting state, by the same specialist. Barefoot height was measured using a portable stadiometer (HR-001, TANITA, Tokyo, Japan). Body mass (BM) and composition were measured using a bioelectrical impedance analyzer (MC-780MA S, TANITA, Tokyo, Japan). The body mass index (BMI) was calculated as BM (kg)/height^2^(m^2^). In addition, fasted resting energy expenditure (REE) was measured by indirect calorimetry using a mobile spiroergometric system (METAMAX 3B-R2, CORTEX Biophysik GmbH, Leipzig, Germany). Before each test, the equipment was calibrated according to the manufacturer’s recommendations. Participants were placed in a supine position in a thermoneutral environment (22–25 °C room temperature) for 45 min before starting the measurements. After reaching a steady state, their O_2_ consumption and CO_2_ production, normalized for temperature, barometric pressure, and humidity, were recorded and averaged at one-minute intervals for 20–45 min and averaged over the entire measurement period. Resting energy expenditure was then calculated from VO_2_ consumption (L.min^−1^) using Weir’s equation [17].

Thereafter, young rugby players participated in a follow-up of an intensive training phase during a pre-season micro-cycle (7 days) at their training center (Montferrand Sports Association-Rugby Section, France). A weekly training load (15 h/week total) included rugby field training (8 h/week), wrestling (4 h/week), and strength and conditioning training (3 h/week). During the follow-up, participants were instructed to consume food at will in their habitual catering facilities (school and training center canteen). All foods were directly weighed by the investigators before and after consumption using an electronic food scale and a nutritional assessment of the dietary intake was performed using the French food composition database (ANSES, 2020). The database was supplemented with details of local foods. The analysis involved the calculation of energy intake (in kcal), macronutrients (carbohydrate [CHO], fat, and protein in grams and proportion) as well as micronutrients. We accounted for the use of CHO/protein supplements in our calculations. Sports nutrition recommendations for adolescents [15], which include nutrition guidelines for health [18], and more recent recommendations [19,20] were used to assess the adequacy of the reported macronutrient intakes. Physical activity energy expenditure (PAEE) was estimated using tri-axial accelerometery (GT3X+, Actigraph LLC, Pensacola, FL, USA). The manufacturer’s guidelines were followed to position the accelerometer on the dominant hip, using an elastic band to secure it over the anterior spine of the iliac crest and align it with the anterior axillary line. During the follow-up period, participants were instructed to wear the device continuously (during and outside training). Data were analyzed in 30 s epochs using the manufacturer’s software (Actilife 6.0, Actigraph LLC, Pensacola, FL, USA). MET intensity thresholds were determined based on previous calibration studies and the cut-points from Evenson et al. [21], which have already been used in young athletes, were chosen because of their higher accuracy compared to other published cut-points [21,22].

### 2.3. Statistical Analysis

Statistical analyses were performed using IBM SPSS Statistics (version 23). Graphing and visualization were carried out using Prism 9 (GraphPad, San Diego, CA, USA) and R studio (version 4.0.5, RCore Team, Boston, MA, USA). Descriptive statistics are presented as mean ± standard deviations and inferences drawn at a 0.05 alpha level. A priori cluster analysis was performed on the dataset containing body fat percentage (BF, %), fat-free mass (BFF, kg), weight (kg), and the player’s position. Continuous variables (BF, BFF, and weight) were normalized using the Z-score method to ensure comparability across scales. K-means clustering was employed to partition the data into distinct groups. The optimal number of clusters was determined using two methods: the elbow method (within-cluster sum of squares, WCSS) [23] and the average silhouette method [24]. Both methods were implemented to ensure robustness in determining the optimal cluster count. The characteristics of the resulting clusters are presented, including age, position, height, weight, energy balance, and dietary intake (both macro and micronutrients). Descriptive statistics were calculated for each variable within each cluster to summarize their profiles. Paired sample t-tests were performed to examine differences between groups.

## 3. Results

### 3.1. Participants Characteristics and K-Means Clustering

Anthropometric characteristics are presented in Table 1 for the whole sample, as well as the two obtained clusters according to body, weight, composition (BF%, FFM), and playing position. Each cluster presented distinct characteristics with cluster 1 (n = 27) predominantly including BW players (85%), having significantly lower weight and body fat (%) compared to cluster 2 (*p* < 0.001 for both). In this cluster, four FWs, particularly from the second row, were identified. These players are characterized by their exceptional height yet present a low BF%. Cluster 2 is by contrast composed mainly of FWs and presented higher body mass (*p* < 0.001). Fat-free mass was slightly higher in cluster 2, although not significantly different from cluster 1 (*p* = 0.1). No significant differences were found between the two clusters in term of age and height.

### 3.2. Energy Balance

**Figure 1:**
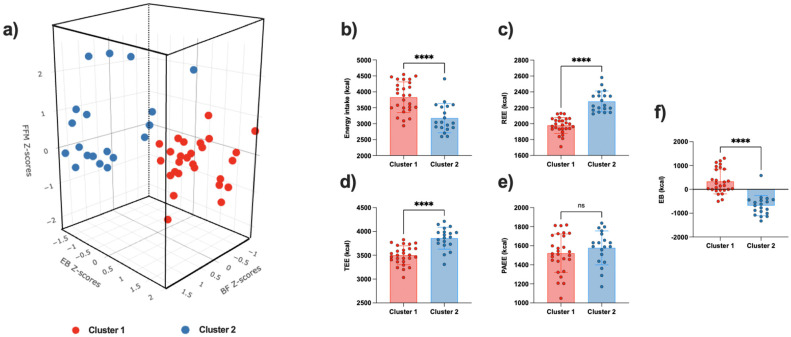
Energy balance components among participants by cluster. (**a**) Three-dimensional representation of the two clusters obtained based on playing position and Z-scores for body mass, FFM, and BF, (**b**) Energy intake, (**c**) Resting energy expenditure, (**d**) Total energy expenditure, (**e**) Physical activity energy expenditure, (**f**) Energy balance. Red dots represent individual data from cluster 1, Blue dots represent individual data from cluster 2. BF: Body fat; EB: Energy balance; FFM: Fat-free mass; PAEE: Physical activity energy expenditure; REE: Resting energy expenditure; TEE: Total energy expenditure; **** indicates *p* < 0.01, ns indicates not significant *p* > 0.05.

Figure 1a illustrates a three-dimensional representation of the two clusters obtained based on playing position and Z-scores for body mass, FFM, and BF. Cluster 1 is distinguished by a positive EB Z-score, in contrast to cluster 2, which displays a negative EB Z-score. This observation was corroborated by the comparison of EB components within each cluster (Figure 1b–f). In fact, there were no significant differences in PAEE between the two clusters, suggesting that they were engaged in a comparable training volume and had a similar level of physical activity during the micro-cycle. However, cluster 2 exhibited a higher TEE, mainly attributed to an elevated REE associated with greater body mass. By contrast, EI was significantly higher in cluster 1 compared to cluster 2 (∆ = 1013.27 kcal, *p* < 0.001). Consequently, cluster 1 was in a positive EB, whereas cluster 2 was in a negative EB (330 ± 517 kcal versus −683 ± 425 kcal, respectively, *p* < 0.001).

### 3.3. Macronutrients and Micronutreints

**Figure 2:**
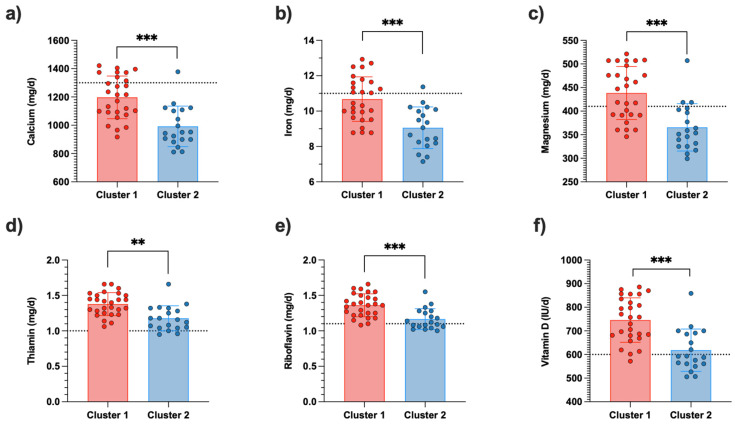
Micronutrients intake among participants by cluster. (**a**) Calcium intake, (**b**) Iron intake, (**c**) Magnesium intake, (**d**) Thiamin intake, (**e**) Riboflavin intake, (**f**) Vitamin D intake. Red dots represent individual data from cluster 1, Blue dots represent individual data from cluster 2. Dotted lines indicate the recommended dietary allowance (RDA) and the sport dietitians Australia position statement for the adolescent athletes; the dotted lines represent the Recommended Dietary Allowances (RDA) in accordance with the position of Sports Dietitians Australia (SDA) for adolescent athletes; *** *p* < 0.001, ** *p* < 0.01.

Table 2 and Figure 2 illustrate the intake of macronutrients and micronutrients in young rugby players, categorized by clusters. Cluster 2 showed a lower intake of all macronutrients when adjusted for body mass, particularly CHO intakes, which was −2.69 g/kg lower compared to cluster 1. Although there was a lower fat intake (−0.47 g/kg) among cluster 2, intakes of polyunsaturated fatty acids (PUFA), saturated fatty acids (SFA), and the ratio of PUFA to SFA (P/S ratio) was not different between the two clusters. In addition, the fiber intake was significantly lower in Cluster 2 compared to Cluster 1 (−8.28 g, *p* = 0.002).

The intake of all micronutrients reported in Figure 2 was significantly lower in cluster 2 than in cluster 1. However, intakes of the evaluated B-group vitamins (thiamin and riboflavin) met the RDA in both clusters (Figure 2d,e). In contrast, intakes of calcium, iron, and magnesium were below the RDA, particularly in Cluster 2 (Figure 2a–c). While daily vitamin D intake met the RDA in Cluster 1, several participants in Cluster 2 did not meet the RDA for this nutrient (Figure 2f).

## 4. Discussion

This study aimed to evaluate the energy balance and dietary intake of young rugby players competing at the national level during a pre-season micro-cycle. While earlier findings indicated that young players were at high risk of insufficient energy intake during intensive training periods [5], the present study provides a more nuanced understanding of young rugby players’ EB, accounting for within-individual differences. We identified two clusters with distinct characteristics and energy balance states, suggesting that young rugby players adapted their dietary intake to align with their body composition goals. The first cluster is characterized by players with a low BF% and predominantly consists of back players, with the exception of four FWs from the second row. This cluster exhibited a positive EB, which suggests an objective of increasing muscle mass. Conversely, the second cluster comprises mainly FWs players and is distinguished by a higher body fat percentage (BF%). This cluster showed a negative energy balance, suggesting that the primary body composition goal is focused on reducing fat mass. Although our study provides more optimistic results than previous ones regarding the risks of an insufficient energy intake in young rugby players, we emphasize the importance of rigorous nutritional assistance, particularly for young players aiming to lose weight, in order to avoid severe caloric restriction during this pivotal period of life, as well as the downstream effects of such practices on their nutritional status.

Rugby is still regarded as one of the most inclusive sports in terms of body types [11]. However, young rugby athletes must meet the demands of the sport and adapt to meet performance standards, as well as size and body composition requirements. The perception that a larger body is crucial for success in rugby is widely held. The game’s rules are continually evolving, now permitting competition for the ball on the ground after a tackle and allowing more substitutions. These changes have enabled teams to select larger players who can engage in physical collisions and to bring in new players when these bigger athletes become fatigued [25]. Consequently, longitudinal studies have shown that players’ body mass increased by 24.3% between 1955 and 2015 [26]. These changes are believed to be driven by the development of professional infrastructure within clubs, supported by the evolving laws of the game. Significantly, professional structures, which exist from junior levels, have allowed players to access full-time team environments, optimizing training and nutrition to enhance muscle mass and develop the strength and power necessary for performance starting from early adolescence. Moreover, rugby is an exceptionally demanding sport that necessitates high levels of strength, speed, power, agility, and both anaerobic and aerobic fitness [1]. Therefore, young rugby players typically engage in cross-training, incorporating diverse types of exercise into their training regimens. Fitness adaptations occur regardless of a player’s body weight, and nutritional strategies should be tailored to each player’s goals. Some young athletes may possess higher body fat percentages yet still develop strength, and their cardiovascular systems will adapt to aerobic exercise during adolescence. It is arguable that body fat could benefit some rugby players (e.g., FWs such as props) by making them heavier and harder to displace, providing impact protection, and adhering to the principle that greater mass can facilitate moving mass, potentially enhancing collision force [27]. However, the detrimental effects of excessive body fat on rugby performance are increasingly considered. Therefore, the current body composition standard involves maximizing muscle mass while maintaining essential body fat to enhance performance. Consequently, modern players have become notably leaner, fitter, and more muscular. Advances in rugby have greatly contributed to this body composition standard evolution. At the elite level, players have slimmed down significantly, with fewer instances of overtly overweight individuals compared to previous decades. This trend is observable across all levels of the sport, from amateur to international, and independently of position or ethnicity [11,28,29,30,31]. Interestingly, it has been suggested that high FFM, low BF%, as well as low skinfold thickness, could predict selection to higher playing standards in young [28] and adult [32] rugby players. The optimal body composition for young rugby players remains undetermined and may vary depending on their playing position. However, it appears that young players recognize the importance of these standards and adjust their energy intake accordingly to improve their chances of achieving these body composition standards. 

Previous research conducted during the pre-season in professional Australian football athletes reported that dietary intakes of these athletes were influenced by a body composition assessment which is also a current practice in rugby. Jenner et al. (2018) argued that players with high body fat percentages (BF%) often restricted carbohydrate intake to achieve their goals surrounding a body composition assessment [33]. This finding is supported by the results of the current study. Absolute CHO was significantly lower in cluster 2 (∆ = −131.51 g). Importantly, along with growth and maturation and rugby-specific demands, pre-season micro-cycles involve intensified training, including a combination of resistance-focused gym workouts, conditioning exercises, and field sessions, and thus normally imply increased energy and nutrients requirements. While it is well established that the CHO requirements increase as the exercise volume increases to restore muscle glycogen [34], previous reports suggest that adolescent athletes do not adjust their nutrients intake to the demands of the training load or different training sessions [35]. Importantly, CHO-dependent energy systems (aerobic and anaerobic) are used in rugby [36]. Moreover, muscle glycogen has been shown to be reduced by ~40% during academy matches [29]. Maximizing muscle glycogen pre-exercise through sufficient CHO intakes is therefore crucial [37,38]. Interestingly, young players may benefit from focusing on the recommendations of “fuel for the work required”—whereby CHO availability is adjusted in accordance with the demands of the upcoming training session(s)—by ensuring key training sessions and competitions are undertaken with sufficient CHO availability so as to promote performance and recovery [39]. The CHO daily intake of players included in cluster 2 (3.91 ± 1.53 g/kg/day) was far below the minimum recommended daily level for fuel and recovery. Adolescent sports nutrition guidelines suggest 6–10 g/kg/day for 1–3 h per day of training [15]. Although this raises the question of whether this amount of CHO is beneficial for players trying to lose weight, the threshold of CHO cutting during periods of high training levels to reduce body fat without detrimental effects on health and performance remains to be determined.

In addition to having a negative energy balance and a lower than recommended CHO intake, our results show that cluster 2 might be at a higher risk of micronutrient deficiencies (i.e., calcium, iron, magnesium, vitamin D, all of which were lower in cluster 2 compared to cluster 1 despite similar PAEE). Importantly, the position statement from Sports Dietitians Australia emphasizes the critical importance of ensuring adherence to the reference values for key micronutrients, notably iron (11 mg/d), calcium (1300 mg/d), and vitamin D (5 mg/d), since deficiencies in these nutrients can result in significant health problems, such as iron deficiency anemia, iron status disorders, and osteoporosis, later in life [15,16]. Moreover, recent recommendations on hypocaloric diets for overweight adolescents suggest reducing energy intake to 500 kcal below the usual spontaneous diet by excluding foods with the highest energy density, such as sugary and fatty foods and high-calorie beverages, and favoring foods with a high nutrient density to overcome micronutrient deficiencies [40]. However, some young rugby players exhibited an EB below −1000 kcal with insufficient intakes of some essential micronutrients such as calcium, iron and magnesium. A severe energy deficit can lead to low energy availability (LEA), which can be detrimental to the athlete’s bone, endocrine, and metabolic health. In addition, in a previous study conducted among the same population, we showed that LEA may be associated with impaired sleep quality, which can negatively affect performance and increase the risk of injury [41]. Consequently, athletes may need support to continue with performance-based nutritional plans, especially those aimed at reducing body fat mass.

This study examines the energy balance (EB) and dietary intake of young rugby players during the pre-season, accounting for within-individual differences and providing new insights into this area. The study’s strengths include its ecological setting and a robust evaluation of energy intake (EI). Additionally, the use of accelerometry allowed us to include a significant sample size compared to previous studies and accounted for overall physical activity energy expenditure (PAEE) both during and outside of training. However, it is important to acknowledge certain limitations. While accelerometry enabled the inclusion of a larger sample size, this method may underestimate energy expenditure, particularly in a sport like rugby. While DXA and doubly labeled water are regarded as the gold standards for assessing body composition and energy expenditure, respectively, they are costly and technically challenging. Future research could enhance accuracy by utilizing DXA for body composition assessments and integrating at least heart rate monitors with accelerometers for better energy expenditure estimation. Furthermore, future studies should investigate whether providing nutritional assistance and enhancing nutrition knowledge can improve the dietary intake and nutritional status of young rugby players. Evaluating the impact of tailored nutrition education programs and personalized dietary interventions could provide valuable insights into optimizing athletes’ health and performance. Longer longitudinal studies could track the long-term dynamic changes of the EB and body composition adaptation (i.e., over a year) and open the horizon for determining the optimal threshold of an energy deficit to achieve the desired body composition without detrimental effects on health in this particular population. The benefits of these initiatives could potentially lead to the development of better practices for nutritional support by stakeholders managing adolescent athletes with high energy expenditure. This would be particularly valuable during periods of significant physical development and high-intensity sports demands where changes in body composition and optimal performance are required.

## 5. Conclusions

The results of the present study show that adolescent male rugby players meet the required protein and fat intakes during a micro-cycle of pre-season training, but their CHO intakes were at the lower limit of international recommendations. Furthermore, the pre-season period is considered the optimal time for players to increase their training volume to achieve meaningful changes in body composition. Our analysis revealed the existence of two clusters with a different EB status, suggesting different dietary strategies, with cluster 1—average 13% of BF—aiming to increase their BM and cluster 2—average 23% of BF—aiming to reduce fat mass. Although changes in body composition are critical to achieve high levels of athletic performance, it is imperative that various dietary factors be considered to prevent any negative impact on the health of young rugby players. Particular attention and nutritional support should be given to young rugby players attempting to reduce fat mass during this critical period of life.

## Figures and Tables

**Table 1:** Participants’ characteristics.

	Total (*n* = 46)	Cluster 1 (*n* = 27)	Cluster 2 (*n* = 19)	*p*
Age (years)	16.17 (0.87)	16.23 (0.85)	15.9 (0.72)	NS
Height (cm)	181.02 (6.69)	181.68 (7.26)	179.07 (4.45)	0.1
Body mass (kg)	82.23 (11.21)	77.97 (9.34)	92.35 (8.53)	<0.001
BMI (kg/m^2^)	25.12 (3.37)	23.62 (2.42)	28.79 (2.23)	<0.001
BF (%)	15.83 (5.3)	12.87 (2.53)	23.1 (1.6)	<0.001
FFM (kg)	68.84 (7.46)	67.71 (6.87)	71.14 (8.05)	0.1
Playing position	19 FW (41%), 27 BW (59%)	4 FW (15%), 23 BW (85%)	15 FW (79%), 4 BW (21%)	-

BF: body fat; BMI, body mass index; BW: Backs players; FFM: fat-free mass; FW: Forward players.

**Table 2:** Macronutrients intake among participants by cluster.

	Total (*n* = 46)	Cluster 1 (*n* = 27)	Cluster 2 (*n* = 19)	*p*
PROT (g)	157.20 (38.84)	163.14 (42.15)	148.73 (32.79)	0.219
CHO (g)	437.46 (158.85)	491.77 (153.21)	360.26 (135.88)	0.004
FAT (g)	131.26 (39.73)	134.51 (38.76)	126.63 (41.68)	0.513
PROT_BM_ (g/kg)	1.95 (0.57)	2.20 (0.57)	1.60 (0.32)	<0.001
CHO_BM_ (g/kg)	5.49 (2.23)	6.59 (1.96)	3.91 (1.53)	<0.001
FAT_BM_ (g/kg)	1.62 (0.54)	1.81 (0.54)	1.34 (0.40)	0.003
PUFA (g)	5.09 (1.16)	5.11 (0.99)	5.06 (1.39)	0.904
SFA (g)	12.07 (1.28)	12.15 (1.41)	11.95 (1.08)	0.618
P/S ratio	0.43 (0.11)	0.43 (0.09)	0.43 (0.13)	0.894
Fiber (g)	35.50 (9.08)	38.92 (7.88)	30.63 (8.58)	0.002

Abbreviations: CHO, carbohydrates; PROT, protein; BM, body mass.

## Data Availability

The data presented in this study are available on request from the corresponding author.
